# Are pediatricians responsible for maintaining high MMR vaccination coverage? Nationwide survey on parental knowledge and attitudes towards MMR vaccine in Serbia

**DOI:** 10.1371/journal.pone.0281495

**Published:** 2023-02-16

**Authors:** Goranka S. Loncarevic, Aleksa Lj Jovanovic, Milena S. Kanazir, Darija B. Kisic Tepavcevic, Gorica D. Maric, Tatjana D. Pekmezovic

**Affiliations:** 1 Institute of Public Health of Serbia, Belgrade, Serbia; 2 Institute of Epidemiology, University of Belgrade Faculty of Medicine, Belgrade, Serbia; Brigham Young University, UNITED STATES

## Abstract

**Aim:**

To assess parental knowledge and attitudes related to MMR vaccination and to determine factors associated with parental decision whether to vaccinate their child with MMR vaccine in Serbian population.

**Methods:**

The selection of participants was performed using multi-phase sampling. Seventeen out of the total 160 public health centers on the territory of Republic of Serbia were randomly selected. All parents of children up to the age of 7 who visited the pediatrician at the public health centers from June to August 2017 were recruited. Parents filled in an anonymous questionnaire regarding their knowledge, attitudes and practices in immunization with MMR vaccine. The relative contribution of different factors was explored by univariable and multivariable logistic regression analysis.

**Results:**

The majority of parents were female (75.2%), with mean age of 34.3 ± 5.7 years, and the average age of children was 4.7 ± 2.4 years, 53.7% of them were girls. In the multivariable model, getting information on vaccination from a pediatrician was associated with 7.5 fold increased probability to vaccinate child with MMR vaccine (OR = 7.52; 95% CI 2.73–20.74; p<0.001), while previous vaccination of the child increased this chance two times (OR = 2.07; 95% CI 1.01–4.27; p = 0.048), and having two children was associated with 84% increase in chance of vaccinating child with MMR vaccine compared to having one child or three or more children (OR = 1.84; 95% CI 1.03–3.29; p = 0.040).

**Conclusion:**

Our study emphasized the key role of pediatricians in the formation of parental attitude on MMR vaccination of their child.

## Introduction

Measles is a contagious, vaccine-preventable disease caused by a virus of the *Paramyxoviridae* family, genus *Morbillivirus*. The disease can lead to serious complications including encephalitis and pneumonia. Globally, more than 140,000 deaths due to measles were registered in 2018, with the predominance of children younger than five years [1]. At the same time, data showed that in 2018, approximately 86% of children worldwide were immunized against measles at the age of one year [1]. The COVID-19 pandemic has posed an additional risk of measles outbreaks bearing in mind that 41 countries have postponed measles campaigns due to pandemic [2].

In Serbia, MMR vaccine is administered in a two dose schedule; first dose at 12 to 15 months of age and second at 7 years, before enrollment in primary school and the vaccination takes place at community health centers. During the period 2015–2019, a total of 75 measles outbreaks were registered in Serbia with a total of 3,608 measles cases [3]. Majority of outbreaks occurred in 2018 with 3,006 measles cases. MMR vaccination coverage for the same period (2015–2019) ranged from 81.0 to 93.4% for the first dose and from 87.5 to 91.9 for the second dose [4]. The distribution of total measles outbreaks and total measles cases along with MMR vaccination coverage in Serbia in the abovementioned time interval is summarized in S1 Table.

Having in mind that various factors (concerns regarding vaccine effectiveness and safety, trust in sources of information, demographic characteristics, etc.) [5] are related to MMR vaccination uptake, the aim of the present study was to assess parental knowledge and attitudes related to vaccination and to determine factors associated with the parental decision whether to vaccinate their child with MMR vaccine in Serbian population.

## Material and methods

A cross-sectional study was conducted in the period June–August 2017. The study was approved by Ethics Committee of the Faculty of Medicine, University of Belgrade (No. 29/XII-9).

### Selection of participants

The selection of participants was performed using multi-phase sampling. A total of 160 health centers distributed along 25 administrative districts are included in primary health care activities of the Republic of Serbia. The study was carried out in four phases. The first phase included all 160 health centers as a cluster. In the second phase, the cluster was divided into subclusters based on population size: the city of Belgrade, Vojvodina and central Serbia, each of which represented a third of the total sample size. In the third phase, health centers were randomly selected and were included in the research based on the principle of 10% of the sample from each selected stratum. In Belgrade, primary health care is provided by 16 health centers in 16 municipalities, from which 2 were randomly selected. In the territory of Vojvodina primary health care is provided by 45 health centers, from which 3 were selected by random selection. In the territory of central Serbia 12 out of 115 health centers were selected by random selection. The calculation of the required sample size was based on a type I error of 5%, for a confidence level of 95%, with an assumed vaccination prevalence of 50%. According to the mentioned criteria, the required sample size was 385 respondents. The total number of selected health centers was 17 (Novi Sad, Subotica, Niš, Sombor, Čačak, Vranje, Užice, Valjevo, Kragujevac, Požarevac, Kraljevo, Ćuprija, Zaječar, Pirot, Šabac and two in Belgrade). The fourth stage of sample formation consisted of all parents of children up to the age of 7 who visited the pediatrician on the day of the examination at the health center. Every parent that agreed to participate signed informed consent.

### Measurement and instrument

The questionnaire used in this study was designed based on data found in literature [6, 7]. The first and the second part of the questionnaire referred to the parents’ demographic characteristics of the parents (age, gender, education level, employment and marital status, monthly income, place of residence (a village was considered as a small community in a rural area; a city was considered a populated area with fixed borders and local self-government), number of children, as well as data on MMR vaccination status, and data on whether they had measles in the past) and of their children (age, gender, birth order, data on premature birth, body weight at birth and information on whether the child had been diagnosed with some health condition so far). Next section of the questionnaire contained 20 questions about vaccination (effects of vaccination, number of doses, age at vaccination, immunization schedule, vaccine-preventable diseases, vaccination following exposure etc.). Section “Attitudes” was divided into 15 statements about parental opinion on vaccination of children with different mandatory and recommended vaccines, and about their sources of information on immunization. Two final parts of the questionnaire were related to reasons for and against vaccination with MMR vaccine using a 5-point Likert scale. Parents filled in only one of those tables depending on their child’s MMR vaccination status.

### Statistical analysis

In the descriptive analysis, means and standard deviation were used for continuous variables, while frequencies and percentages were used for nominal data. Internal consistency of the reasons for MMR vaccination and against it was determined using Cronbach’s alpha and values above 0.7 were considered acceptable. Construct validity of reasons for and against MMR vaccination was evaluated using factor analysis with varimax rotation. The cut-off for eigenvalue was set at 1.0. Finally, the relative contribution of different factors to MMR vaccination status was explored by univariate and multivariate logistic regression analysis. In these analyses, the dependent variable was MMR vaccination status of the child. All variables that reached statistical significance in the univariate analyses were then analyzed together in a multivariate model with the same dependent variable. For the purposes of the univariate and multivariate logistic regression analyses, new variable “Previous vaccination of the child” was computed combining variables “My child /children have been vaccinated against tuberculosis (BCG vaccine)”, “My child /children have been vaccinated against diphtheria, tetanus and pertussis with vaccines provided in the immunization schedule”, “My child /children have been vaccinated against hepatitis B”, “My child / children have been vaccinated against polio”, and “My child / children have been vaccinated against *Haemophilus influenzae* type b diseases”. The child was labeled previously vaccinated if it had received all of the previously scheduled vaccines and not vaccinated if it hadn’t received any of the scheduled vaccines. P value less than 0.05 was considered statistically significant. All analyses were performed using SPSS (Statistical package for Social Sciences), version 17.0.

## Results

Demographic characteristics of the parents and children included in the study are shown in Table 1. The majority of parents were female (75.2%), with mean age of 34.3 ± 5.7 years, married (87.3%), employed (78.9%), living in a town (82.4%) with total monthly income between 50.000 and 100.000 Serbian Dinars (37.7%) and with secondary school education level (43.3%). Highest frequency was observed in parents having two children (53.2%), being vaccinated with MMR (76.8%) and not having had measles before (53.3%) (Table 1). The average age of children was 4.7 ± 2.4 years, 53.7% of them were girls. Furthermore, 57.4% of children were the firstborn child. Premature birth was registered in 2.8%. The average birth weight was 3379.8 ± 512.4 grams and some health disorder was diagnosed in 1.8% of the study sample (Table 1).

**Table 1:** Demographic and clinical characteristics of study participants.

Parents			Children		
Variable	N	%	Variable	N	%
Sex			Sex		
Male	141	24.8	Male	263	46.3
Female	427	75.2	Female	305	53.7
Age	34.3 ± 5.7 ^a^	18–51^b^	Age	4.69 ± 2.37 ^a^	0–14 ^b^
Marital status			Birth order		
Married	496	87.3	First	326	57.4
Divorced	36	6.3	Second	201	35.4
Extramarital union	19	3.3	Third	38	6.7
Widowed	3	0.5	Fourth	3	0.5
Never married	14	2.5			
Employment status			Premature birth		
Employed	448	78.9	Yes	16	2.8
Unemployed	120	21.1	No	552	97.2
Education			Gestation week of birth (if born prematurely)		
Primary school	20	3.5	29	1	0.2
			30	3	0.5
Secondary school	246	43.3	31	3	0.5
			32	4	0.7
			33	2	0.4
College	67	11.8			
			34	1	0.2
Bachelor’s degree	200	35.2			
			36	2	0.4
Master’s degree	27	4.8	Total	16	2.8
PhD	8	1.4			
Place of residence			Health disorder at birth		
Town	468	82.4	Yes	10	1.8
Village	100	17.6	No	558	98.2
Total number of children			Diagnosis		
One	202	35.6	Heart diseases	1	0.1
Two	302	53.2	Lung diseases	5	0.5
Three	54	9.5	Kidney diseases	1	0.1
Four and more	10	1.7	Other	3	0.3
Monthly income (family budget)			Body weight at birth (grams)	3379.82 ± 512.41^a^	1200–5170 ^b^

^a^ mean ±SD,

^b^ range

The distribution of correct vaccination knowledge answers is presented in S2 Table. The greatest proportion of correct answers was obtained for the following statements: “There are several different vaccines” (98.4%), “Vaccination may be temporarily postponed in case of fever” (98.2%), and “Vaccination prevents diseases” (97.9%). On the other hand, less than half of participants (47.2%) knew that “Unvaccinated children in the collective are protected from diseases by vaccinated children”. Low proportion of correct answers was also registered for the statements “A sick child can be vaccinated to alleviate the severity of the disease” (63.2%) and “Newborns should not receive the hepatitis B vaccine” (64.8%).

The majority of parents agreed that their children have been vaccinated against tuberculosis (BCG vaccine) (96.8%), diphtheria, tetanus and whooping cough (95.4%), hepatitis B (96.1%) and polio (96.8%) (S3 Table). Parents least agreed with the statement “Are you getting vaccinated against the flu?” (16.5%) and “I am informed about vaccination through the media and the internet” (39.8%) (S3 Table).

Exploratory factor analysis of MMR vaccination acceptance and refusal scales revealed five different domains of MMR vaccination acceptance scale (explaining 61.733% of the variance) and six domains in MMR vaccination refusal scales (explaining 74.816% of the variance) (Table 2 and S4 and S5 Tables). The reliability of MMR vaccination-refusal and -acceptance scales is presented in S6 Table. Both scales demonstrated good reliability (Cronbach’s alpha >0.7) as well as their subscales.

**Table 2:** Exploratory factor analysis of the reasons for not receiving the MMR vaccine for pre-school children.

Reasons	Mean score	Concerns about the MMR vaccine’s effectiveness and safety	Values and comfort	Knowledge of the disease and the vaccine	Absence of the pediatrician’s recommendation	Unavailability of the MMR vaccine	Distrust of the vaccine
I am worried about my child getting measles / mumps / rubella after vaccination	3.3 ± 1.2	0.170	0.031	0.007	-0.229	0.137	**0.863** ^a^
I believe that immunity after a natural infection is better than vaccination	3.3 ± 1.6	**0.831** ^a^	0.216	0.040	-0.002	0.119	0.125
I am worried about side effects after vaccination	3.6 ± 1.4	**0.741** ^a^	0.128	-0.152	-0.131	-0.177	0.128
I have never seen a child get measles/ mumps / rubella	2.9 ± 1.5	0.387	-0.059	-0.070	0.254	-0.542	**0.562** ^a^
I am afraid of the child’s reaction to the needle and / or pain	2.1 ± 1.3	0.073	**0.740** ^a^	0.230	0.146	-0.152	-0.039
I don’t have enough time to take my child for vaccinations	1.9 ± 1.2	0.401	-0.094	0.165	**0.621** ^a^	0.408	0.045
Religious beliefs do not allow me to vaccinate my child	1.8 ± 1.2	0.006	**0.694** ^a^	0.124	0.597	0.038	0.050
My child’s pediatrician advised me not to vaccinate my child	1.7 ± 1.1	0.008	0.198	0.056	**0.833** ^a^	0.111	-0.166
I don’t think the MMR vaccine is safe enough	3.0 ± 1.5	**0.857** ^a^	-0.016	0.173	0.081	0.076	-0.010
I don’t have enough knowledge about measles, mumps, and rubella	3.0 ± 1.2	0.050	0.117	**0.939** ^a^	0.134	-0.014	-0.059
I don’t have enough knowledge about the measles, mumps, and rubella vaccine	2.8 ± 1.2	0.042	0.164	**0.930** ^a^	0.032	0.036	0.026
I think that measles, mumps, and rubella are not serious diseases	2.6 ± 1.3	**0.624** ^a^	-0.103	0.419	0.068	0.229	-0.423
Crowds and waiting in health centers discourage me from bringing my child for vaccination	2.4 ± 1.4	**0.479** ^a^	0.404	0.185	-0.008	0.033	-0.050
A person I really appreciate has not vaccinated his/her children	2.2 ± 1.6	**0.596** ^a^	0.237	0.154	0.445	-0.253	-0.132
The MMR vaccine is not mandatory for children	2.8 ± 1.7	0.433	**0.548** ^a^	-0.265	-0.065	0.020	0.035
The MMR vaccine causes autism and neurological disorders in children	2.9 ± 1.6	**0.782** ^a^	0.096	-0.026	0.197	0.110	0.164
The cost of traveling to a health facility limits me from bringing my child	1.5 ± 0.8	-0.005	**0.711** ^a^	0.148	0.058	0.516	0.043
The MMR vaccine was not available at the Health Center, so I could not vaccinate the child	1.4 ± 1.0	0.176	0.023	-0.031	0.240	**0.802** ^a^	0.088
Vaccines impair the body’s resistance	2.0 ± 1.3	**0.698** ^a^	-0.182	-0.057	0.233	0.092	0.373

^a^ Bold values indicate the highest loading weights

Univariable logistic regression analysis revealed that significant predictors of a child’s MMR vaccination status are families with two children (OR = 1.93; 95% Confidence Interval (CI) 1.13–3.28; p = 0.016), living in town (OR = 0.55; 95% CI 0.31–0.97; p = 0.037), parent being vaccinated against measles, mumps and rubella (OR = 1.45; 95% CI 1.06–2.00; p = 0.019), parental total vaccination knowledge score (OR = 1.34; 95% CI 1.20–1.49; p<0.001), previous vaccination of the child (OR = 3.65; 95% CI 1.99–6.71; p<0.001) and getting information on vaccination from a pediatrician (OR = 15.74; 95% CI 6.23–39.76; p<0.001) (Table 3). When these variables were analyzed together in multivariable model, getting information on vaccination from a pediatrician was associated with 7.5 fold increased probability to vaccinate child with MMR vaccine (OR = 7.52; 95% CI 2.73–20.74; p<0.001), while previous vaccination of the child increased this chance two times (OR = 2.07; 95% CI 1.01–4.27; p = 0.048). Additionally, having two children was associated with 84% increase in chance of vaccinating child with MMR vaccine compared to having one child or three or more children (OR = 1.84; 95% CI 1.03–3.29; p = 0.040). Finally, higher total vaccination knowledge score of parents increased by 23% the chance of the child receiving the MMR vaccine (OR = 1.23; 95% CI 1.09–1.39; p<0.001) (Table 3).

**Table 3:** Predictors of child’s MMR vaccination status.

Variables	Unadjusted models			Adjusted model		
	OR	95% CI	p	OR	95% CI	p
Parental age (years)	1.03	0.98–1.08	0.207			
Gender	1.20	0.68–2.14	0.532			
Female vs. Male						
Marital status	0.80	0.31–2.09	0.652			
Married/cohabiting vs. others						
Education level	0.87	0.52–1.47	0.601			
Secondary school vs. higher education						
Employment status	0.60	0.33–1.06	0.080			
Employed vs. unemployed						
Number of children	1.93	1.13–3.28	**0.016** ^a^	1.84	1.03–3.29	**0.040** ^a^
Two vs. others						
Monthly income	1.39	0.79–2.42	0.252			
50,000–100,000 vs. others						
Place of residence	0.55	0.31–0.97	**0.037** ^a^	0.72	0.38–1.36	0.312
Village vs. town						
Parental MMR vaccination status	1.45	1.06–2.00	**0.019** ^a^	0.91	0.63–1.32	0.609
Yes vs. no						
Parental measles infection	1.38	0.94–2.04	0.099			
Yes vs. no						
Premature child birth	1.94	0.25–14.94	0.524			
Yes vs. no						
Presence of health condition in child	0.50	0.10–2.42	0.390			
Yes vs. no						
Total vaccination knowledge score	1.34	1.20–1.49	**<0.001** ^a^	1.23	1.09–1.39	**<0.001** ^a^
Previous vaccination of the child	3.65	1.99–6.71	**<0.001** ^a^	2.07	1.01–4.27	**0.048** ^a^
Yes vs. no						
Getting information on vaccination from a pediatrician	15.74	6.23–39.76	**<0.001** ^a^	7.52	2.73–20.74	**<0.001** ^a^
Yes vs. no						
Getting information on vaccination from media and internet	0.68	0.40–1.14	0.142			
Yes vs. no						

^a^ Bold values indicate statistical significance

## Discussion

In our study, 96% of parents reported pediatricians as their source of information on immunization. This clearly implicates that the pediatrician’s recommendation plays a pivotal role in the parents’ final decision on child’s complete and age-appropriate immunization. The finding is crucial because it indicates that both vaccine-hesitant and non-hesitant parents respected advice obtained from pediatricians.

It has already been demonstrated that the confidence of health-care workers (HCWs) in the benefits and safety of vaccines correlates with their educational level [8]. Educational level of HCWs significantly varies depending on the curriculum of undergraduate studies [9, 10]. A project performed at the University of Munich (Germany), identified that the most effective principle in studying immunization is the competency-based-curriculum, which integrates all of the important learning objectives about immunization into a single teaching program, instead of students traditionally studying it superficially inside various subjects like microbiology, epidemiology, immunology, infectious diseases, pediatrics, and public health [11]. Furthermore, the need for educational activities in medical and paramedical curricula on vaccines and vaccination programs in Europe has recently been emphasized by the European Joint Action on Vaccination (EU-JAV) [12, 13]. Therefore, one of the urgent strategies should be to raise awareness among pediatricians by increasing their evidence-based knowledge on MMR vaccine effectiveness and safety profile, as well as their awareness of the professional and ethical responsibility they have as the primary source of information for parents.

As in our study, others have shown that recommendation obtained from pediatrician is most commonly stated as the reason for immunization acceptance in general population, and more significantly, the absence of recommendation is frequently stated as the reason for non-vaccination [8, 14, 15]. More precisely, vaccine-hesitant pediatricians do not guide hesitant parents while considering childhood immunization [16].

Another important issue is the pediatricians’ professional and ethical responsibility, which also demands their additional and continuous education in this regard, in order to develop scientifically appropriate awareness and beliefs regarding their role in maintaining a successful immunization program in the population.

Parents use a variety of sources of information on vaccination ranging from the one received from pediatricians, official healthcare authorities, and scientific papers, to books, magazines, websites, social media and opinions and attitudes of different laypersons [8, 15]. In the recently published study in Switzerland, the child’s pediatrician was perceived as the most trusted source of information (71% of vaccine-hesitant parents vs. 85% of non-hesitant) [17]. However, vaccine-hesitant parents more frequently stated sources other than the pediatrician compared to non-hesitant parents, such as social networks (43% of vaccine-hesitant parents vs. 28% non-hesitant parents), books, magazines and websites that criticize official vaccination recommendations (21% vs. 0%), healthcare workers other than pediatrician (21% vs. 13%), and personal gut feeling (3% vs. 1%) [17]. An important observation is that vaccine-hesitant parents had less trust in pediatricians and public health authorities, and were less satisfied with their healthcare providers compared to non-hesitant parents, which could have led to them turning to alternative sources of information [17].

A study evaluating predictors of MMR vaccination status had not been conducted previously in the Republic of Serbia. Having in mind the fact that the recommended vaccination coverage is extremely high (≥95%), it is clear that even a minor decrease in vaccination coverage will result in an increased probability for measles outbreaks to occur. Before 2011, MMR vaccination coverage in Serbia was above the desired threshold of immunization with values 96–97%; after that values started to decrease with the lowest coverage reported in 2016 (81%) [4].

In Athens, Greece, in a sample of 3,399 children attending public nurseries the MMR vaccination coverage was only 63.7% (completely immunized) compared to all other vaccines from national immunization schedule, with coverage above 94% [6].

The main strength of our study is the representative sample at the national level. Limitations refer to the cross-sectional study design which complicates determination of causal relationship, as well as self-reporting of data collected by means of the questionnaire which could have introduced information bias.

Our study emphasized the key role of pediatricians in the formation of parental attitude on MMR vaccination of their child. Based on the results of our study, we believe that substantial changes in immunization policy are necessary. Key elements of the improved program should include the improvement of undergraduate and postgraduate medical curriculum in the field of immunization and continual parental education in the community. Additionally, by identifying the specific predictors of the decision not to vaccinate children against measles, mumps and rubella, a specific profile of parents could be created. If the parents’ profiles are available, the HCWs can tailor their approach to each individual category of parents in order to decrease vaccination hesitancy and by doing so increase the coverage.

## Supporting information

S1 Table Number of measles outbreaks with total number of measles cases and MMR vaccination coverage (%) in Serbia in the period 2015–2019. (DOC)

S2 Table Percentages of correct vaccination knowledge answers. (DOC)

S3 Table The statements of parents regarding vaccination. (DOC)

S4 Table Exploratory factor analysis of the reasons for receiving the MMR vaccine for pre-school children. (DOC)

S5 Table Percentage of the variance explained of the vaccination acceptance and vaccination-refusal related factors. (DOC)

S6 Table The reliability of MMR vaccination-refusal and -acceptance scales. (DOC)
