# Supplementary material for: Are pediatricians responsible for maintaining high MMR vaccination coverage? Nationwide survey on parental knowledge and attitudes towards MMR vaccine in Serbia
Source: PLoS One. 2023 Feb 16;18(2):e0281495. doi: 10.1371/journal.pone.0281495 (PMC9934397; doi:10.1371/journal.pone.0281495)
Supplement: S1 Table — (DOC) [file pone.0281495.s001.doc]

Supplementary Table S1 Number of measles outbreaks with total number of measles cases and MMR vaccination coverage (%) in Serbia in the period 2015-2019

|  | 2015 | 2016 | 2017 | 2018 | 2019 |
| --- | --- | --- | --- | --- | --- |
| No. of outbreaks | 11 | 2 | 17 | 44 | 1 |
| Total measles cases | 214 | 19 | 366 | 3,006 | 3 |
| MMR 1^st^ dose coverage (%) | 84.0 | 81.0 | 85.2 | 93.4 | 88.0 |
| MMR 2^nd^ dose coverage (%) | 87.5 | 91.1 | 91.6 | 91.6 | 91.9 |
