# Supplementary material for: Are pediatricians responsible for maintaining high MMR vaccination coverage? Nationwide survey on parental knowledge and attitudes towards MMR vaccine in Serbia
Source: PLoS One. 2023 Feb 16;18(2):e0281495. doi: 10.1371/journal.pone.0281495 (PMC9934397; doi:10.1371/journal.pone.0281495)
Supplement: S2 Table — (DOC) [file pone.0281495.s002.doc]

Supplementary Table S2 Percentages of correct vaccination knowledge answers

| Statements | Correct answer | |
| --- | --- | --- |
|  | N | % |
| 1. Vaccination prevents diseases | 556 | 97.9 |
| 2. There are several different vaccines | 559 | 98.4 |
| 3. Vaccination is a safe procedure | 532 | 93.7 |
| 4. Vaccination is carried out at all ages | 475 | 83.6 |
| 5. Vaccines can cause adverse events | 492 | 86.6 |
| 6. Vaccines must be stored at a certain temperature, and must not be frozen | 528 | 93.0 |
| 7. The schedule of immunization in Serbia is unique for children of a certain age | 547 | 96.3 |
| 8. Vaccination may be temporarily postponed in case of fever | 558 | 98.2 |
| 9. Permanent delay in the administration of a vaccine is extremely rare | 513 | 90.3 |
| 10. Unvaccinated children in the collective are protected from diseases by vaccinated children | 268 | 47.2 |
| 11. Vaccination begins at the maternity hospital | 545 | 96.0 |
| 12. Newborns in Serbia will start immunization against tuberculosis at birth | 522 | 91.9 |
| 13. Newborns should not receive the hepatitis B vaccine | 368 | 64.8 |
| 14. The age when the children are vaccinated was chosen so that they are protected from certain infectious diseases in the best manner, and at the earliest possible time. | 542 | 95.4 |
| 15. The prescribed number of doses of vaccines is needed in order for the child to be completely protected | 549 | 96.7 |
| 16. According to the compulsory schedule of immunization, children receive the last dose of the vaccine in the final grade of primary school | 482 | 84.9 |
| 17. Diseases against which children are vaccinated can be very serious, accompanied by complications and can be fatal | 544 | 95.8 |
| 18. If an unvaccinated child becomes ill, it can infect other children and adults who are not protected | 508 | 89.4 |
| 19. A sick child can be vaccinated to alleviate the severity of the disease | 359 | 63.2 |
| 20. Unvaccinated children can get sick later in life and have complications of the disease that could have been prevented by vaccination | 496 | 87.3 |
