# Supplementary material for: Are pediatricians responsible for maintaining high MMR vaccination coverage? Nationwide survey on parental knowledge and attitudes towards MMR vaccine in Serbia
Source: PLoS One. 2023 Feb 16;18(2):e0281495. doi: 10.1371/journal.pone.0281495 (PMC9934397; doi:10.1371/journal.pone.0281495)
Supplement: S3 Table — (DOC) [file pone.0281495.s003.doc]

Supplementary Table S3 The statements of parents regarding vaccination

| Statements | Yes | No |
| --- | --- | --- |
| My child /children have been vaccinated against tuberculosis (BCG vaccine) | 550  (96.8%) | 16  (2.8%) |
| My child /children have been vaccinated against diphtheria, tetanus, and whooping cough with vaccines provided in the immunization schedule | 542  (95.4%) | 26  (4.6%) |
| My child /children have been vaccinated against hepatitis B | 546  (96.1%) | 22  (3.9%) |
| My child / children have been vaccinated against polio | 550  (96.8%) | 18  (3.2%) |
| My child / children have been vaccinated against *Haemophilus influenza* type b diseases | 502  (88.4%) | 66  (11.6%) |
| My child / children have been vaccinated against measles, mumps, and rubella (MMR vaccine) | 502  (88.4%) | 66  (11.6%) |
| I obtain information regarding vaccination from my child’s pediatrician | 545  (96%) | 23  (4%) |
| After he/she experienced a side-effect, I would give other vaccines to my child | 429  (75.5%) | 139  (24.5%) |
| I would vaccinate my child with the chickenpox vaccine on the recommendation of my child’s pediatrician | 459  (80.8%) | 109  (19.2%) |
| I would vaccinate my child against rotaviral diseases on the recommendation of my child’s pediatrician | 468  (82.4%) | 100  (17.6%) |
| I would vaccinate my child against human papilloma virus (HPV) disease on the recommendation of my child’s pediatrician | 445  (78.3%) | 123  (21.7%) |
| Vaccination is the most effective measure in preventing the disease | 517  (91.0%) | 51  (9.0%) |
| I would recommend / motivate others to vaccinate their children | 503  (88.6%) | 65  (11.4%) |
| I obtain information regarding vaccination through the media and the internet | 226  (39.8%) | 342  (60.2%) |
| Vaccinated against the flu | 94  (16.5%) | 474  (83.5%) |
