# Supplementary material for: Are pediatricians responsible for maintaining high MMR vaccination coverage? Nationwide survey on parental knowledge and attitudes towards MMR vaccine in Serbia
Source: PLoS One. 2023 Feb 16;18(2):e0281495. doi: 10.1371/journal.pone.0281495 (PMC9934397; doi:10.1371/journal.pone.0281495)
Supplement: S4 Table — (DOC) [file pone.0281495.s004.doc]

Supplementary Table S4 Exploratory factor analysis of the reasons for receiving the MMR vaccine for pre-school children

| Reasons | Mean score | Protection of the child, and its surroundings | Comfort, effectiveness, and official recommendation | Social influence | Fear of complications of the diseases | Severity of the disease |
| --- | --- | --- | --- | --- | --- | --- |
| I did not want my child to get sick from these diseases | 4.6 ± 1.0 | **0.733** ^a^ | 0.145 | 0.086 | 0.078 | -0.095 |
| The child has a chronic illness | 1.5 ± 1.2 | -0.003 | -0.100 | 0.158 | -0.195 | **0.583** ^a^ |
| I think that measles, mumps, and rubella are serious diseases | 4.5 ± 0.9 | **0.682** ^a^ | 0.293 | -0.003 | 0.172 | -0.062 |
| I do not want my child to pass the disease onto other children | 4.4 ± 1.0 | **0.858** ^a^ | 0.100 | 0.104 | 0.060 | 0.075 |
| I don't want my child to pass the disease onto family members | 4.3 ± 1.1 | **0.867** ^a^ | 0.079 | 0.127 | 0.071 | 0.035 |
| I am encouraged by family members | 3.4 ± 1.6 | 0.223 | 0.125 | **0.694** ^a^ | 0.082 | 0.213 |
| I am encouraged by the media | 2.5 ± 1.5 | 0.034 | -0.002 | **0.866** ^a^ | 0.003 | 0.218 |
| I received information about the importance of vaccination against these diseases from the media | 2.9 ± 1.5 | 0.031 | 0.108 | **0.815** ^a^ | -0.043 | 0.082 |
| I think the vaccine is safe and effective | 4.4 ± 0.9 | 0.360 | **0.715** ^a^ | 0.037 | 0.016 | -0.078 |
| The vaccine is free | 4.3 ± 1.3 | 0.083 | **0.663** ^a^ | 0.219 | 0.027 | 0.213 |
| Vaccination is mandatory | 4.6 ± 0.8 | 0.094 | **0.717** ^a^ | 0.042 | 0.211 | -0.069 |
| The chosen doctor advised me to vaccinate my child | 4.7 ± 0.7 | 0.073 | **0.543** ^a^ | 0.073 | 0.328 | -0.075 |
| I am afraid that I will be absent from work due to my child's illness | 2.5 ± 1.6 | -0.032 | 0.178 | 0.203 | 0.031 | **0.706** ^a^ |
| My family members were sick with these diseases as children | 2.3 ± 1.4 | -0.023 | -0.042 | 0.136 | 0.235 | **0.603** ^a^ |
| The vaccine will improve the child's health | 4.3 ± 1.0 | 0.361 | **0.543** ^a^ | -0.179 | 0.130 | 0.370 |
| Measles can cause complications in diseased children | 4.5 ± 0.8 | 0.410 | 0.268 | -0.190 | **0.458** ^a^ | 0.320 |
| Rubella can cause miscarriage during pregnancy | 4.4 ± 0.9 | 0.077 | 0.092 | 0.049 | **0.860** ^a^ | 0.061 |
| Mumps can cause complications in older age | 4.6 ± 0.8 | 0.197 | 0.313 | 0.000 | **0.783** ^a^ | -0.033 |

^a^ Bold values indicate the highest loading weights
