# Supplementary material for: Are pediatricians responsible for maintaining high MMR vaccination coverage? Nationwide survey on parental knowledge and attitudes towards MMR vaccine in Serbia
Source: PLoS One. 2023 Feb 16;18(2):e0281495. doi: 10.1371/journal.pone.0281495 (PMC9934397; doi:10.1371/journal.pone.0281495)
Supplement: S5 Table — (DOC) [file pone.0281495.s005.doc]

Supplementary Table S5 Percentage of the variance explained of the vaccination acceptance and vaccination-refusal related factors

| Vaccination refusal | | | Vaccination acceptance | | |
| --- | --- | --- | --- | --- | --- |
| Factors | Eigen value | Percentage of variance explained | Factors | Eigen value | Percentage of variance explained |
| Concerns about the MMR vaccine’s effectiveness and safety | 5.499 | 28.943 | Protection of the child, and its surroundings | 4.796 | 26.643 |
| Values and comfort | 2.906 | 15.294 | Comfort, effectiveness, and official recommendation | 2.406 | 13.364 |
| Knowledge of the disease and the vaccine | 1.755 | 9.238 | Social influence | 1.655 | 9.197 |
| Absence of the pediatrician’s recommendation | 1.575 | 8.292 | Fear of complications of the diseases | 1.189 | 6.606 |
| Unavailability of the MMR vaccine | 1.322 | 6.958 | Severity of the disease | 1.066 | 5.923 |
| Distrust of the vaccine | 1.157 | 6.091 |  |  |  |
| Total percentage of the variance explained | 74.816 | | Total percentage of the variance explained | 61.733 | |
