# Supplementary material for: Are pediatricians responsible for maintaining high MMR vaccination coverage? Nationwide survey on parental knowledge and attitudes towards MMR vaccine in Serbia
Source: PLoS One. 2023 Feb 16;18(2):e0281495. doi: 10.1371/journal.pone.0281495 (PMC9934397; doi:10.1371/journal.pone.0281495)
Supplement: S6 Table — (DOC) [file pone.0281495.s006.doc]

Supplementary Table S6 The reliability of MMR vaccination-refusal and -acceptance scales

| Vaccination refusal | | Vaccination acceptance | |
| --- | --- | --- | --- |
| Factors | Cronbach’s alpha | Factors | Cronbach’s alpha |
| Concerns about the MMR vaccine’s effectiveness and safety | 0.874 | Protection of the child, and its surroundings | 0.839 |
| Values and comfort | 0.672 | Comfort, effectiveness, and official recommendation | 0.706 |
| Knowledge of the disease and the vaccine | 0.945 | Social influence | 0.781 |
| Absence of the pediatrician’s recommendation | 0.647 | Fear of complications of the diseases | 0.723 |
| Unavailability of the MMR vaccine | / | Severity of the disease | 0.458 |
| Distrust of the vaccine | 0.512 |  |  |
| Total | 0.851 | Total | 0.796 |
